# Effects of Highly Absorbable Curcumin in Patients with Impaired Glucose Tolerance and Non-Insulin-Dependent Diabetes Mellitus

**DOI:** 10.1155/2019/8208237

**Published:** 2019-11-23

**Authors:** Masafumi Funamoto, Kana Shimizu, Yoichi Sunagawa, Yasufumi Katanasaka, Yusuke Miyazaki, Hideaki Kakeya, Hajime Yamakage, Noriko Satoh-Asahara, Hiromichi Wada, Koji Hasegawa, Tatsuya Morimoto

**Affiliations:** ^1^Division of Molecular Medicine, School of Pharmaceutical Sciences, University of Shizuoka, Shizuoka 422-8526, Japan; ^2^Clinical Research Institute, National Hospital Organization Kyoto Medical Center, Kyoto 612-8555, Japan; ^3^Department of System Chemotherapy and Molecular Sciences, Division of Bioinformatics and Chemical Genomics, Graduate School of Pharmaceutical Sciences, Kyoto University, Kyoto 606-8501, Japan

## Abstract

Oxidative stress is enhanced by various mechanisms. Serum oxidized low-density lipoprotein (LDL) is a useful prognostic marker in diabetic patients with coronary artery disease. To examine the effects of Theracurmin®, a highly absorbable curcumin preparation, on glucose tolerance, adipocytokines, and oxidized LDL, we conducted a double-blind placebo-controlled parallel group randomized trial in patients with impaired glucose tolerance or non-insulin-dependent diabetes mellitus. We randomly divided the patients with impaired glucose tolerance or non-insulin-dependent diabetes mellitus and stable individuals into the placebo group and the Theracurmin® (180 mg daily for 6 months) group. Of the 33 patients analyzed, 18 (14 males and 4 females) were administered placebo and 15 (9 males and 6 females) were administered Theracurmin®. The patient characteristics did not differ between the two groups. The primary endpoint, HbA1c, did not differ significantly between the two groups. However, the level of *α*1-antitrypsin-low-density lipoprotein (AT-LDL), the oxidized LDL, significantly increased (*p* = 0.024) in the placebo group from the beginning of the trial up to 6 months, although there was no such change in the Theracurmin® group. The percentage change in BMI from the beginning of the trial up to 6 months tended to be higher in the Theracurmin® group than in the placebo group. Patients in the Theracurmin® group tended to have a larger percentage change in adiponectin and LDL-C than those in the placebo group. Patients in the Theracurmin® group showed a smaller percentage change in AT-LDL than those in the placebo group. This study suggests that the highly absorbable curcumin could potentially inhibit a rise in oxidized LDL in patients with impaired glucose tolerance or non-insulin-dependent diabetes mellitus. This trial is registered with UMIN000007361.

## 1. Introduction

With abrupt increases in obesity and changes in lifestyle and their effect on global health and economics, type 2 diabetes has become increasingly prevalent worldwide [1]. Adiponectin, an adipokine derived from adipose tissue [2], improves glucose and lipid metabolism and prevents atherosclerosis [3–5]. Obesity reduces the production/secretion and action of adiponectin in adipose tissue resulting in insulin resistance and a concomitant increase in the risk of diabetes mellitus and atherosclerosis [6, 7].

Curcumin ((1*E*,6*E*)-1,7-bis(4-hydroxy-3-methoxyphenyl)1,6-heptadiene-3,5-dione), a polyphenol with anti-inflammatory and antioxidant activities, is found naturally in turmeric. Previous studies have reported beneficial effects of curcumin [8, 9]. In two rat models of chronic heart failure following hypertensive heart disease and myocardial infarction, curcumin was effective in inhibiting the progression of heart failure [10]. A diet containing curcumin fed for 6 weeks to obese db/db mice improved their impaired glucose tolerance [11]. A clinical study in which 240 patients with prediabetes were randomly divided into two groups, one receiving placebo and the other receiving curcumin (250 mg/day) for 9 months, reported that 16.4% of the patients receiving placebo, but none of those receiving curcumin, developed type 2 diabetes [12].

Despite its immense clinical importance, curcumin usage is limited due to low absorbability when ingested and poor bioavailability. Therefore, we used a drug delivery system (DDS) to develop a curcumin preparation (Theracurmin®) that was highly absorbable by the intestinal tract. In an aqueous solution, natural curcumin forms extremely large granules, while Theracurmin® contains extremely fine granules of curcumin with a coating. This DDS ensures that curcumin is consistently dispersed in an aqueous solution and thus improves its absorption by the intestinal tract [13].

In diabetes, oxidative stress is enhanced by various mechanisms including the generation of advanced glycation end products, autoxidation of glucose, and disorders of polyol metabolism [14]. Reactive oxygen species, generated in the process of glycation [15], produce oxidized LDL [16, 17]. The level of serum oxidized LDL has been shown to be a useful predictive marker of cardiovascular events in patients with type 2 diabetes [18]. *α*1-Antitrypsin-low-density lipoprotein (AT-LDL), a recently identified oxidized low-density lipoprotein, is a complex that promotes atherosclerosis. We previously reported that Theracurmin® reduced levels of AT-LDL in patients with chronic obstructive pulmonary disease [19]. The levels of AT-LDL in human serum and at sites of atherosclerotic lesions indicate the activity of foam cells in these lesions [16, 17]. However, the effects of curcumin on glucose tolerance, adipocytokines, and oxidized LDL in patients with impaired glucose tolerance or non-insulin-dependent diabetes mellitus remain unknown. Thus, we conducted a double-blind parallel group randomized trial with Theracurmin® and placebo to examine the effects of curcumin on glucose tolerance, adipocytokines, and oxidized LDL in patients with impaired glucose tolerance or non-insulin-dependent diabetes mellitus.

## 2. Materials and Methods

### 2.1. Patients

We fully explained the purpose of this trial both verbally and in writing to potential subjects. Male or female patients aged between 20 and 85 years were enrolled in this trial following the provision of voluntary written consent. The participating patients were required to stop smoking at least 4 months prior to the initiation of the trial. Patients with impaired glucose tolerance or non-insulin-dependent diabetes mellitus were required to be in a stable condition, i.e., having HbA1c levels between 6.3% and 8.4%, with HbA1c levels maintained at <8.0% for at least 3 months prior to administration of the preparation.

### 2.2. Exclusion Criteria

We excluded patients if they were diagnosed with any of the following conditions, if their laboratory results indicated any of the following conditions, or who were deemed to meet any of the following criteria by the investigator:

1. Patients with type 1, type 2, or secondary diabetes receiving insulin therapy
2. Patients with severe renal dysfunction (serum creatinine of ≥4.0 mg/dL) or patients on dialysis
3. Patients with severe liver dysfunction or cirrhosis
4. Patients with unstable angina, acute myocardial infarction, or severe coronary artery disease (left main stem or 3-vessel disease)
5. Patients with severe cardiovascular dysfunction (e.g., shock, heart failure, or myocardial infarction) or severe pulmonary dysfunction (e.g., pulmonary embolism) or patients with any other condition likely to cause hypoxemia
6. Patients who had developed a cerebrovascular disorder (e.g., cerebral hemorrhage, cerebral infarction, or subarachnoid hemorrhage) up to 3 months prior to providing consent
7. Patients with severe ketosis or in a diabetic precoma or coma
8. Patients having a severe infection, patients awaiting or recovering from surgery, or patients undergoing severe trauma
9. Patients who were malnourished, starving, or debilitated or had pituitary or adrenal insufficiency
10. Patients with an active malignancy
11. Patients with persistent anemia (defined as Hb of ≤6.0 mg/dL)
12. Patients regularly receiving oral or injectable corticosteroids
13. Patients allergic to the trial supplement
14. Patients who were pregnant or nursing or wished to conceive
15. Other individuals deemed unsuitable for this trial by their primary physician (e.g., those with poor compliance)

### 2.3. Trial Protocol

The Ethics Committee of the Kyoto Medical Center reviewed and approved this trial. We registered the trial with UMIN (UMIN: 000007361) before commencement. Based on the philosophy of the ethical principles originating in the Declaration of Helsinki, we ensured the protection of the rights and welfare of our patients. We ensured the scientific nature and reliability of this trial and its safety by conducting it in accordance with the principles of Good Clinical Practice and strictly adhering to the Ministry of Health, Labor, and Welfare's Ethical Guidelines for Clinical Research. In this double-blind parallel group randomized trial, the trial preparations (Theracurmin® (already sold as a health supplement) and placebo) were randomly numbered and their identities were concealed. Enrolled patients were instructed to ingest the preparation in a capsule form (90 mg in 3 capsules, 30 mg per capsule) once after breakfast and once after dinner (twice a day, i.e., 180 mg per day) for 6 months. Our primary endpoint was HbA1c level. Enrolment in and commencement of the trial took place between March 2012 and April 2014.

### 2.4. Statistical Analysis

Parametric (continuous) data are expressed as the mean ± standard deviation (SD), and nonparametric data are expressed as the median (minimum, maximum). We used the unpaired *t*-test to compare the parametric data between the two groups and Mann-Whitney *U* test to compare the nonparametric data. For analysis of ≥3 matched groups, we analyzed the parametric data using repeated measures ANOVA and then performed an unpaired *t*-test as a *post hoc* test. We analyzed the nonparametric data using Friedman's test and then performed a Wilcoxon signed-rank test as a *post hoc* test. We analyzed the trend test using Jonckheere-Terpstra test (nonparametric data). We carried out all analyses using SPSS version 22.0 for Windows (IBM Japan, Ltd., Tokyo, Japan).

## 3. Results

### 3.1. Basic Characteristics of Participants

Of the 52 patients enrolled in this trial, 19 dropped out or discontinued for reasons including rash on the left arm (1 patient), tinnitus (1 patient), melena (1 patient), soft stools (1 patient), voluntary withdrawal (5 patients), choking sensation (1 patient), admission for surgery (4 patients), admission for a dosage reduction (1 patient), constipation (1 patient), initiation of chemotherapy (1 patient), and addition or modification of antidiabetic drugs (2 patients). Melena in 1 patient receiving Theracurmin® might have been related to the trial supplement, but the other reasons for dropout or discontinuation were unrelated to the trial supplement. Hence, we analyzed 33 patients consisting of 18 (14 males and 4 females) receiving placebo and 15 (9 males and 6 females) receiving Theracurmin®. Patient characteristics prior to receiving placebo or Theracurmin® are presented in Table 1. The two groups did not differ in terms of sex, age, BMI, SBP, DBP, HbA1c, BS, TG, LDL-C, HDL-C, UA, *γ*-GTP, Cre, SAA-LDL, AT-LDL, adiponectin, leptin, hs-CRP, or antidiabetic medicines as noted in Table 1.

### 3.2. AT-LDL Was Increased in the Placebo Group but Not Increased in the Theracurmin® Group

We compared the parameters prior to intervention (placebo or Theracurmin®) and at 3 and 6 months postintervention (Tables 2 and 3). The primary endpoint (HbA1c) did not change significantly in the placebo or Theracurmin® groups. BS as well as AT-LDL was significantly increased (*p* = 0.017 (BS), *p* = 0.024 (AT-LDL)) in patients receiving placebo at 3 and 6 months after commencement of the trial, but it did not change significantly in those receiving Theracurmin®. LDL-C did not differ between patients receiving Theracurmin® or placebo for 6 months. Both TG and *γ*-GTP decreased significantly (*p* = 0.015 and *p* = 0.007, respectively) in patients receiving Theracurmin® between 3 months and 6 months after receiving that preparation, but not in those receiving placebo. Thereafter, we examined whether there were time-dependent changes for each item. In the placebo group, a significant time-dependent increase in AT-LDL levels was observed before administration, at three months after administration, and at six months after administration (Table 2, *p* = 0.017). However, there were no time-dependent changes in AT-LDL levels in the Theracurmin® group (Table 3).

### 3.3. Adiponectin Tended to Be Positive in the Theracurmin® Group

We compared the percentage change in the individual parameters after intervention for 6 months (Table 4). Compared to the patients receiving placebo, those receiving Theracurmin® had a larger percentage change in their BMI. The percentage change in adiponectin, which decreases as a result of weight gain, was negative in patients receiving placebo, whereas it was positive in those receiving Theracurmin® who tended to gain weight. Compared to patients receiving placebo, those receiving Theracurmin® tended to have a larger percentage change in LDL-C but a smaller percentage change in AT-LDL.

## 4. Discussion

BMI was higher in patients receiving Theracurmin® than in those receiving the placebo. While “good” adiponectin has antiatherosclerotic action and usually decreases with obesity, adiponectin tended be higher in patients receiving Theracurmin® when compared to those receiving placebo. Curcumin has been shown to inhibit the expression of inflammatory cytokines, such as monocyte chemoattractant protein-1 and tumor necrosis factor-alpha in white adipose tissue, and increase the expression of adiponectin, resulting in antiatherosclerotic action [20, 21]. Curcumin also induces the conversion of white adipose tissue to brown adipose tissue in mice [22]. Thus, the effects of curcumin on fat presumably led to an increase in adiponectin level. In a study that investigated the effects of various treatments on the secretion of adiponectin in 3T3-L1 adipocytes, pioglitazone treatment increased the secretion of adiponectin by 2.5-fold, whereas curcumin treatment increased this secretion by 1.3-fold [23]. Curcumin is less effective than PPAR*γ* agonists and may not have led to a significant increase in adiponectin levels, likely due to the small sample size in the present study.

Traditionally, spice turmeric has been used to increase appetite [24]. A study that showed that curcumin causes weight gain in rats is supported by another report implicating it in weight gain in patients with colorectal cancer [25, 26]. Similarly, in the current trial, BMI tended to be higher in patients receiving Theracurmin® than in those receiving the placebo. Studies have also suggested that curcumin may increase appetite [27, 28], although the precise mechanism of this action is still unclear.

A meta-analysis of the effects of curcumin on leptin suggests that curcumin may decrease leptin levels [27]. The present study also showed that in the Theracurmin® group, from baseline, leptin demonstrated a decreasing trend six months after administration (*p* = 0.084). When the body weight increases, leptin resistance naturally increases and its concentration in the blood also increases. In the present study, subjects in the Theracurmin group showed a higher propensity to gain weight than those in the placebo group (*p* = 0.077). It is with high possibility that this is caused by the improvement in leptin resistance upon the treatment of curcumin.

Postprandial hyperglycemia suggests that vascular endothelial cells might have been damaged by oxidative stress. Ceriello et al. used meal tolerance tests to compare a carbohydrate-rich diet and a low-carbohydrate diet and reported that levels of oxidized LDL and malondialdehyde, a marker of lipid peroxidation, increased 2 h after eating [29]. Human umbilical vein endothelial cells cultured on a medium with a high glucose concentration showed increased evidence of oxidative stress and apoptosis, suggesting hyperglycemia may damage vascular endothelial cells [30]. In patients receiving placebo, LDL-C level did not change but oxidized LDL level increased significantly, suggesting that an increase in blood glucose may cause *α*1-antitrypsin oxidation via reactive oxygen species, resulting in an increase in AT-LDL levels. Curcumin exhibits antioxidant activity because of the conjugated double bonds and the two phenolic hydroxyl groups in the molecule, allowing elimination of reactive oxygen species [31, 32]. The antioxidant activity might be the mechanism by which curcumin inhibits oxidized LDL.

Theracurmin® has been reported to exert the same effects as curcumin in vitro. In cultured cancer cells such as PC3, DU145, and LNCaP, Theracurmin demonstrated the same level of anticancer activity at the same concentration as curcumin [33]. Therefore, we believe that DDS treatment to create Theracurmin® does not affect curcumin activity.

### 4.1. Limitations

The limitations of this trial were the small sample size and the short duration of the intervention (6 months). Therefore, it is premature to come to a clear conclusion regarding the beneficial effects of curcumin on adiponectin and oxidized LDL in patients with impaired glucose tolerance and non-insulin-dependent diabetes mellitus. A large-scale and long-term study in the future with cardiovascular events as the primary endpoint is expected.

## 5. Conclusions

The current trial demonstrated that in patients with impaired glucose tolerance or non-insulin-dependent diabetes mellitus, curcumin inhibited the increase in oxidized LDL. Curcumin may be used in the future to prevent cardiovascular diseases, the most common complication of diabetes. The ability of curcumin to prevent cardiovascular disease needs to be determined in a long-term study using a large sample.

## Figures and Tables

**Table 1:** Baseline characteristics of the participants in each group.

	Placebo	Theracurmin®	*p* value
Sex (M/F)	13/4	9/6	0.450
Age (years)	69 ± 7	70 ± 6	0.857
Antihypertensive drug (% of total)	88.2	86.7	>0.999
*α*-Glucosidase inhibitor (% of total)	23.5	13.3	0.658
DPP-4inhibitor (% of total)	11.8	13.3	>0.999
Sulfonylurea (% of total)	0	13.3	0.212
Biguanide (% of total)	23.5	13.3	0.659
Glinide (% of total)	0	6.7	0.469
Thiazolidinediones (% of total)	5.9	0	>0.999
BMI (kg/m^2^)	25.0 ± 2.6	24.9 ± 4.6	0.951
SBP (mmHg)	123 ± 12	127 ± 18	0.474
DBP (mmHg)	68 ± 10.9	68.7 ± 8.5	0.901
HbA1c (%)	6.1 [6.0, 6.7]	6.1 [6.0, 6.3]	0.775
BS (mg/dL)	110.0 [99.5, 160.5]	109.5 [100.0, 145.8]	0.766
TG (mg/dL)	159 [88, 197]	144 [86, 177]	0.521
LDL-C (mg/dL)	101 ± 21	100 ± 20	0.860
HDL-C (mg/dL)	54 ± 12	63 ± 15	0.063
UA (mg/dL)	5.2 ± 1.5	5.6 ± 1.1	0.366
*γ*-GTP (IU/L)	29.0 [21.5, 49.5]	32.0 [21.0, 57.0]	0.910
Cre (mg/dL)	0.8 [0.8, 0.9]	0.8 [0.6, 0.9]	0.265
SAA-LDL (*μ*g/mL)	5.0 [4.8, 8.0]	5.0 [4.0, 7.0]	0.686
AT-LDL (*μ*g/mL)	1.3 [1.0, 1.4]	1.1 [1.0, 1.3]	0.823
Adiponectin (*μ*g/mL)	8.0 ± 4.0	7.1 ± 2.8	0.528
Leptin (ng/mL)	5.1 [2.3, 7.0]	4.7 [2.1, 15.4]	0.865
hs-CRP (mg/dL)	0.6 [0.4, 1.8]	0.6 [0.2, 1.1]	0.560
CCr (mL/min/1.73m^2^)	77.2 ± 24.9	78.7 ± 21.0	0.857

Data are presented as the mean ± SD, or median [minimum, maximum], or number of patients. M/F: male/female; BMI: body mass index; SBP: systolic blood pressure; DBP: diastolic blood pressure; HbA1c: hemoglobin A1c; BS: blood sugar; TG: triglyceride; LDL-C: low-density lipoprotein cholesterol; HDL-C: high-density lipoprotein cholesterol; UA: uric acid; *γ*-GTP: *γ*-glutamyl transpeptidase; Cre: creatinine; SAA-LDL: serum amyloid A-LDL; AT-LDL: a1-antitrypsin-LDL; hs-CRP: high-sensitivity C-reactive protein; CCr: creatinine clearance.

**Table 2:** Change of each parameter in patients treated with placebo.

Placebo	0M (0 month)	3M	6M	*p* value for trend test	*p* value		
					0M vs. 3M	0M vs. 6M	3M vs. 6M
BMI (kg/m^2^)	25.0 ± 2.6	24.9 ± 2.6	24.8 ± 2.7	0.109	0.716	0.109	0.164
SBP (mmHg)	123.2 ± 12.4	125.4 ± 15.1	125.1 ± 18.1	0.541	0.474	0.541	0.901
DBP (mmHg)	68.3 ± 10.9	69.0 ± 9.5	70.5 ± 13.8	0.454	0.661	0.454	0.508
HbA1c (%)	6.1 [6.0, 6.7]	6.2 [5.9, 6.4]	6.3 [6.1, 6.6]	0.587	0.417	0.886	0.681
BS (mg/dL)	110.0 [99.5, 160.5]	112.0 [95.0, 139.0]	134.0 [109.5, 155.0]	0.850	0.705	0.723	**0.017**
TG (mg/dL)	144.0 [87.0, 198.5]	134.0 [93.8, 206.8]	118.0 [84.3, 232.8]	0.677	0.326	0.326	0.277
LDL-C (mg/dL)	101.9 ± 21.3	95.0 ± 20.3	101.9 ± 26.8	0.992	**0.041**	0.992	0.183
HDL-C (mg/dL)	53.8 ± 12.0	52.5 ± 12.6	52.0 ± 12.9	0.192	0.228	0.192	0.723
UA (mg/dL)	5.2 ± 1.5	5.0 ± 1.1	5.1 ± 1.2	0.545	0.401	0.545	0.684
*γ*-GTP (IU/L)	29.0 [21.5, 49.5]	28.0 [24.0, 49.0]	32.0 [18.5, 55.0]	0.252	0.536	0.244	0.950
Cre (mg/dL)	0.8 [0.8, 0.9]	0.8 [0.7, 1.0]	0.8 [0.7, 1.0]	0.326	0.530	0.348	0.887
SAA-LDL (*μ*g/mL)	5.0 [4.8, 8.0]	6.0 [4.0, 18.8]	5.0 [5.0, 8.5]	0.915	0.174	0.630	0.261
AT-LDL (*μ*g/mL)	1.3 [1.0, 1.4]	1.4 [1.2, 1.6]	1.4 [1.1, 1.7]	**0.017**	0.071	**0.024**	0.942
Adiponectin (*μ*g/mL)	8.0 ± 4.0	7.7 ± 3.8	7.6 ± 3.5	0.302	0.159	0.302	0.706
Leptin (ng/mL)	5.1 [2.3, 7.0]	4.7 [3.3, 7.7]	4.0 [2.2, 6.6]	0.279	0.660	0.221	0.073
hs-CRP (mg/dL)	0.6 [0.4, 1.8]	0.8 [0.5, 4.2]	0.9 [0.5, 1.3]	0.385	0.730	0.778	0.551
CCr (mL/min/1.73m^2^)	69.1 [54.2, 88.9]	74.1 [57.3, 92.7]	77.4 [59.7, 96.1]	0.723	0.569	0.438	0.733

BMI: body mass index; SBP: systolic blood pressure; DBP: diastolic blood pressure; HbA1c: hemoglobin A1c; BS: blood sugar; TG: triglyceride; LDL-C: low-density lipoprotein cholesterol; HDL-C: high-density lipoprotein cholesterol; UA: uric acid; *γ*-GTP: *γ*-glutamyl transpeptidase; Cre: creatinine; SAA-LDL: serum amyloid A-LDL; AT-LDL: a1-antitrypsin-LDL; hs-CRP: high-sensitivity C-reactive protein; CCr: creatinine clearance.

**Table 3:** Change of each parameter in patients treated with Theracurmin®.

Theracurmin®	0M (0 month)	3M	6M	*p* value for trend test	*p* value		
					0M vs. 3M	0M vs. 6M	3M vs. 6M
BMI (kg/m^2^)	24.9 ± 4.6	24.8 ± 4.5	25.0 ± 4.5	0.992	0.307	0.518	0.118
SBP (mmHg)	127.9 ± 18.1	130.1 ± 20.0	129.3 ± 17.8	0.936	0.637	0.744	0.906
DBP (mmHg)	69.7 ± 7.9	68.4 ± 12.3	69.3 ± 11.1	0.746	0.704	0.875	0.801
HbA1c (%)	6.1 [6.0, 6.3]	6.2 [5.9, 6.7]	6.1 [5.9, 6.8]	0.933	0.179	0.552	0.719
BS (mg/dL)	109.5 [100.0, 145.8]	108.0 [99.3, 135.0]	99.0 [91.5, 122.8]	0.130	0.861	0.079	0.124
TG (mg/dL)	144.0 [86.0, 177.0]	147.0 [88.0, 219.0]	120.0 [87.0, 170.0]	0.669	0.245	0.532	**0.015**
LDL-C (mg/dL)	99.5 ± 19.8	102.3 ± 18.8	100.9 ± 19.2	0.992	0.355	0.745	0.719
HDL-C (mg/dL)	62.7 ± 14.8	61.7 ± 14.1	60.2 ± 13.1	0.546	0.509	0.266	0.279
UA (mg/dL)	5.6 ± 1.1	5.6 ± 1.4	5.7 ± 1.1	0.684	0.797	0.438	0.833
*γ*-GTP (IU/L)	32.0 [21.0, 57.0]	33.0 [23.0, 57.0]	33.0 [21.0, 45.0]	0.917	0.239	0.432	**0.007**
Cre (mg/dL)	0.8 [0.6, 0.9]	0.8 [0.7, 0.9]	0.8 [0.7, 0.9]	0.677	0.330	0.161	0.551
SAA-LDL (*μ*g/mL)	5.0 [4.0, 7.0]	6.0 [5.0, 9.3]	5.5 [4.0, 8.8]	0.935	0.223	0.837	0.858
AT-LDL (*μ*g/mL)	1.1 [1.0, 1.3]	1.1 [1.0, 1.4]	1.1 [1.0, 1.3]	0.962	0.720	0.722	0.796
Adiponectin (*μ*g/mL)	7.1 ± 2.8	7.2 ± 2.7	7.4 ± 2.8	0.837	0.800	0.383	0.535
Leptin (ng/mL)	4.7 [2.1, 15.4]	3.8 [2.0, 14.6]	3.2 [2.3, 11.0]	0.588	0.695	0.084	0.075
hs-CRP (mg/dL)	0.6 [0.2, 1.1]	0.5 [0.3, 1.1]	0.5 [0.2, 1.2]	0.797	0.861	0.861	0.917
CCr (mL/min/1.73m^2^)	72.4 [66.7, 95.0]	72.4 [58.6, 90.5]	71.6 [61.3, 90.8]	0.723	0.460	0.074	0.650

BMI: body mass index; SBP: systolic blood pressure; DBP: diastolic blood pressure; HbA1c: hemoglobin A1c; BS: blood sugar; TG: triglyceride; LDL-C: low-density lipoprotein cholesterol; HDL-C: high-density lipoprotein cholesterol; UA: uric acid; *γ*-GTP: *γ*-glutamyl transpeptidase; Cre: creatinine; SAA-LDL: serum amyloid A-LDL; AT-LDL: a1-antitrypsin-LDL; hs-CRP: high-sensitivity C-reactive protein; CCr: creatinine clearance.

**Table 4:** Percentage changes of parameter in placebo and Theracurmin® groups.

	*n*	Placebo	*n*	Theracurmin®	*p* value
*Δ*BMI	17	−1.0 ± 2.4	15	0.3 ± 1.5	0.077
*Δ*SBP	17	1.5 ± 10.4	14	1.8 ± 12.7	0.944
*Δ*DBP	17	-3.1 [-10.7, 19.8]	14	0.0 [-14.7, 9.8]	0.662
*Δ*HbA1c	17	−0.5 ± 5.9	15	1.1 ± 4.2	0.388
*Δ*BS	17	-0.7 [-13.4, 40.8]	14	-5.4 [-17.6, 2.4]	0.219
*Δ*TG	17	-3.7 [-32.6, 13.4]	15	-8.5 [-25.4, 24.0]	0.835
*Δ*LDL-C	17	-3.5 [-13.5, 2.4]	15	1.3 [-8.2, 18.5]	0.220
*Δ*HDL-C	17	−4.3 ± 10.2	15	−2.6 ± 13.1	0.675
*Δ*UA	17	0.1 ± 14.1	15	1.8 ± 8.2	0.688
*Δγ*-GTP	17	3.4 ± 20.4	15	−1.0 ± 18.2	0.531
*Δ*Cre	17	−3.3 ± 12.0	15	3.0 ± 5.9	0.072
*Δ*SAA-LDL	14	6.8 ± 40.9	14	9.0 ± 44.3	0.891
*Δ*AT-LDL	14	14.9 ± 19.3	14	4.6 ± 24.8	0.233
*Δ*adiponectin	14	−3.7 ± 14.5	13	5.1 ± 16.9	0.160
*Δ*leptin	14	−11.6 ± 33.2	13	−13.7 ± 33.9	0.872
*Δ*hs-CRP	14	-1.7 [-27.5, 108.0]	13	-1.2 [-40.3, 42.5]	0.771
*Δ*CCr	17	2.5 [-4.1, 6.3]	15	-2.6 [-7.5, 2.1]	0.168

BMI: body mass index; SBP: systolic blood pressure; DBP: diastolic blood pressure; HbA1c: hemoglobin A1c; BS: blood sugar; TG: triglyceride; LDL-C: low-density lipoprotein cholesterol; HDL-C: high-density lipoprotein cholesterol; UA: uric acid; *γ*-GTP: *γ*-glutamyl transpeptidase; Cre: creatinine; SAA-LDL: serum amyloid A-LDL; AT-LDL: a1-antitrypsin-LDL; hs-CRP: high-sensitivity C-reactive protein; CCr: creatinine clearance.

## Data Availability

The datasets generated during and/or analyzed during the current study are available from the corresponding author on reasonable request.
